# Hints for the Sick-Room

**Published:** 1888-09-29

**Authors:** M. M. Basil

**Affiliations:** Author of "The Commoner Accidents to Life and Limb."


### Hints for the Sick-room.

By M. M. Basil, M.A., M.B., C.M., Author of "The Commoner Accidents to Life and Limb."

ADMINISTRATION OF MEDICINES (*continued*).

For an examination by the finger or touch alone, the simple position on the left side or on the back is to be preferred. Sims' position is be resorted to only when the parts are to be exposed by Sims' speculum or some modification of it.

The different varieties of vaginal douches may be briefly considered in this connexion. Two essentially different objects may be had in view in giving such douches, and it is important to thoroughly understand this difference, so that grave mistakes may be avoided.

Most douches are intended to act on the organs high up in the pelvis. The patient must therefore be so placed as to have the vagina fully dilated with the liquid. This can be secured either by raising the pelvis while the patient lies on the back, or by placing her in Sims' position. The injection may be given either by the irrigator, or much more efficiently by the intermittent action of a syringe worked by the hand. If necessary, very hot water may be used without any dread of scalding the patient, provided that the vaginal-tube is not metallic, and the water does not remain long in contact with the external parts. The vaginal-tube must have side holes and no perforation at the free end, and it should be kept in close contact with the posterior wall of the cavity. These precautions are necessary, to prevent the serious results which would follow the unintentional injection of the douche into the uterus, an accident especially likely to happen in the lying-in condition.

In another class of cases it is particularly desirable that the fluid should not reach the higher portions of the pelvis. The douche is intended here more for the external and lower parts than the pelvic organs. The patient must therefore be placed in such a position that the liquid cannot distend the parts by its gravitating action. The class of cases where it was pointed out that catheterisation in the dark may lead to mischief are also those in which a high vaginal injection may seriously add to the patient's disease.

Before we pass on to the nursing of surgical conditions and of special diseases, we must consider those duties in the clinical observation of every case which are usually performed by the nurse. The extent of work done in this direction varies in each hospital, but in all places some clinical facts can only be observed by the one in constant attendance on the patient. In private work the nurse usually takes a larger share in the observation of the case, and you must therefore be prepared for these duties.

The temperature of every important case of disease is usually taken twice a-day, or oftener. There is no observation in the sick-room which must be made with greater care and intelligence than the recording of the temperature of the patient. It is the one phenomenon in disease which is least likely to lead to deception, and, like all God's truths, it must be observed with scrupulous exactness. A wrong thermometric reading may lead to grievous errors in diagnosis and treatment; the responsibility of the nurse is very great here, and the greater the confidence placed in her the greater must be her effort to give a correct representation of the clinical features of disease, and of the temperature in particular. When the temperature cannot be taken correctly it is better to omit all attempts at doing the work than to perform it in an off-hand or hurried manner. Entire absence of information will not easily mislead anyone, but false information may mislead even the most wary observer.

For ascertaining the temperature of the body correctly, several conditions must be fulfilled. (1) The thermometer must be a fairly correct measurer of heat. This must be ascertained from time to time by comparing it with a standard thermometer, or comparing the temperature of the same patient taken at the same time by a number of thermometers. (2) The thermometer must be in working condition. The index of it especially must be attended to, as, in spite of the assurances of most instrument makers on the imperishable nature of the index of their thermometers, the fact is that until quite recently few thermometers were satisfactory in this respect. The "imperishable index" must have been so called very much on the principle which guided the transformed weaver in his proposal to call his dream "Bottom's Dream" because it had no bottom. (3) The index must be set. This is best done by holding the thermometer firmly at some distance from the bulb, and moving it two or three times rapidly, so as to give the bulb a jerking movement away from the wrist—in fact, shaking the index down towards the bulb. It is recommended by some to hold the thermometer in one hand like a pen, then move the hand downwards rapidly, and have its motion arrested by the other hand. This, however, is not by any means so satisfactory a method as the first one. The index must not be shaken down too violently, otherwise it may be entirely destroyed. (4) The thermometer must be placed properly in the axilla, so that every part of the bulb is in direct contact with the body. The armpit must be dried, else the layer of moisture all around the bulb will modify the reading. The bulb of the thermometer must not be imbedded in a mass of hair, and no scrap of clothing must intervene between it and the body. The thermometer must be placed in the axilla itself; its bulb must not be allowed to go past the space and stick out behind the armpit. In emaciated patients a nest, or hollow space, often remains in the axilla when the arm is simply placed by the side. The bulb must not be placed into this hollow. The arm should be drawn well forward, so as to occupy the side and front of the chest. The insertion of the thermometer must not be entrusted to the patient. (5) The thermometer must be kept in position for a sufficiently long time. When the temperature is taken in the axilla seven and a-half to ten minutes must be allowed; in young children, when the axilla is not easily accessible, the groin may be used with convenience. In reckoning the time you must be guided by the clock, as it is utterly impossible here to form any estimate of time by guessing. These ten minutes are the longest of the kind known in or out of the sick-room. It is necessary to prepare the parts a few minutes before the thermometer is inserted, so as to obtain correct results. In a ward, all patients should have the arm kept by the side and the shoulders well covered some minutes before the time for taking temperatures. The temperature should be taken at or about the same time every day, and when the contrary is the case the hour should be noted down distinctly. (6) There must be no addition made artificially to the degree of fever present. It is seldom that such a trick is resorted to by patients, but, nevertheless, it is well to be aware of the possibility of raising the index of a thermometer by quick and repeated movement of the arm on the chest-walls. (7) To read the thermometer correctly the index must be looked at while the shaft is kept quite straight. If the instrument be looked at in a slanting direction, considerable difference in the reading will be obtained. The thermometric reading must be registered on the chart at once, otherwise no value whatever can be attached to the result. No human being can day after day remember correctly a dozen or more figures which, within the limits of a few degrees, resemble each other very much and yet constantly differ from each other.

*(To be continued.)*

